# Evaluation of risk factor profiles for acute coronary syndrome among Indian adults 

**DOI:** 10.6026/9732063002001396

**Published:** 2024-10-31

**Authors:** Krishna Bhagwan Patil, Dilip Patil

**Affiliations:** 1 Department of Medicine, Krishna Institute of Medical Sciences, Karad - 415110, Maharashtra, India; 2 Department of General medicine, Krishna Institute of Medical Sciences, Karad - 415539, Maharashtra, India

**Keywords:** Blood sugar and lipid level, acute coronary syndrome, ST-elevation myocardial infarction, non-ST elevation myocardial infarction, risk factor, like chest pain, palpitation and sweating, vomiting, giddiness

## Abstract

Acute coronary syndrome (ACS) refers to a group of conditions that include ST-elevation myocardial infarction (STEMI), non-ST elevation myocardial infarction (NSTEMI) and unstable angina. Therefore, it is of interest to assess the risk factor (R/F) of ACS. Hence, 67 patients underwent investigation with the help of history and clinical examination test like electrocardiogram (ECG), blood sugar and lipid level. Data shows R/F was seen more for males than females with 30-40 years of age range. Thus, we conclude that, multiple complications like chest pain, palpitation, sweating, vomiting and giddiness should not be overlooked.

## Background:

Acute coronary syndrome (ACS) encompasses a range of conditions that arise from the abrupt obstruction of blood flow to the heart. It spans from a phase of unstable angina (US-A) that can potentially be reversed to irreversible cell death caused by a myocardial infarction (MI). This issue is of great concern due to the severe impact this illness has on the vibrant lives of young adults. It is increasingly becoming a major cause of death worldwide [1]. ACS remains a significant contributor to morbidity and mortality in hospitals across Western Europe [2]. In the future, south Asian countries are expected to bear more than half of the worldwide burden of cardiovascular disease [3]. In most developed nations, coronary heart disorders are the major cause of death because of their prevalence. At the moment, cardio-vascular disease (CVD) is the leading cause of death in Iran, and CA illnesses are among the conditions that fall under this category. It is important to note that health care is at the top of the list of factors that generate costs [4, 5]. These illnesses are responsible for a substantial amount of morbidity, impairment, and decreased productivity. The number of fatalities caused by heart attacks in the globe is around 29.6% of the overall causes of death. This means that in 2014, heart attacks were responsible for twenty percent of deaths that occurred on the European continent. It is estimated that this complication was responsible for more than 385 thousand fatalities in the United States in 2013 [6]. Research has shown that cardiovascular illnesses are the leading cause of death in Iran, accounting for 45% of all deaths and 26% of all years of life lost. Furthermore, CVD account for 10.4% of the entire burden of diseases [7]. Therefore, it is of interest to report the risk factor (R/F) of ACS.

## Materials and Methods:

The current hospital based cross sectional observational study conducted in Department of General Medicine, Krishna Institute of Medical Sciences, Karad and Maharashtra starting from September 2022 ending to December 2023 with a total of 67 patients. Each patient underwent for detailed history-taking and clinical examination followed with test like 12 lead ECG at administration and whenever necessary and lab investigation which includes cardiac enzyme trop I by AIA 360 TOSOH, blood glucose level by EM 360 TRANSASIA and lipid profile [Total cholesterol (TC), Triglycerides (TG), High-density lipoprotein (HDL), Low-density lipoprotein (LDL)] by XL 640.

## Inclusion criteria:

[1] 18-40 years of age.

[2] Fulfilling the criteria for ACS.

[3] Those who were willing to give consent.

[4] Both genders.

## Exclusion criteria:

[1] Those who declined YTO provide consent.

[2] Below 18 years.

[3] Above 40 years.

## Results:

Table 1 shows that, for individuals aged 18-30 years, there were 27 cases, which account for 40.30% of the total. For those aged 31-40 years, there were 40 cases, making up 59.70% of the total. This data suggests that the prevalence of cases is higher in the 31-40 years age group compared to the 18-30 years age group. Table 2 shows that, among the total cases, 50 were male, which constitutes 74.63% of the cases. On the other hand, 17 cases were female, accounting for 25.37% of the cases. This data indicates significantly higher number of males as compared to females. Table 3 shows that, among individuals aged 18-30, 44.00% reported a SL (11 cases), while 33.33% (14 cases) reported this in the 31-40 age group, totalling 25 cases. Chest pain was prevalent in 52.00% (13 cases) of the younger group and 57.14% (24 cases) of the older group, totalling 39 cases. Palpitation was noted in 62.50% (15 cases) of the younger group and 23.30% (10 cases) of the older group, totalling 25 cases. Sweating affected 56.00% (14 cases) of the younger group and 42.86% (18 cases) of the older group, totalling 32 cases. Giddiness was reported by 24.00% (6 cases) of the younger group and 33.33% (14 cases) of the older group, totalling 20 cases. Vomiting occurred in 40.00% (10 cases) of the younger group and 69.05% (29 cases) of the older group, totalling 39 cases. AP affected 28.00% (7 cases) of the younger group and 30.95% (13 cases) of the older group, totalling 20 cases. Age wise assessment of clinical features revealed significant difference only in symptoms of palpitation and vomiting. Table 4 shows that, for a SL, 42.59% of males (23 cases) and 15.38% of females (2 cases) are affected, totalling 25 cases. Chest pain is reported by 66.67% of males (36 cases) and 23.08% of females (3 cases), with 39 cases overall. Palpitation are experienced by 37.04% of males (20 cases) and 23.08% of females (3 cases), totalling 23 cases. Sweating is noted in 50.00% of males (27 cases) and 38.46% of females (5 cases), with a total of 32 cases. Data shows giddiness affects 25.93% of males (14 cases) and 46.15% of females (6 cases), totalling 20 cases. Vomiting is experienced by 53.70% of males (29 cases) and 76.92% of females (10 cases), with 39 cases in total. AP is reported by 27.78% of males (15 cases) and 38.46% of females (5 cases), totalling 20 cases. Gender wise assessment of clinical features revealed significant difference in symptoms of SL, chest pain, sweating and vomiting.

Table 5 shows that, among the participants, 19.40% (13 individuals) have a normal BMI ranging from 18.5 to 23. A significant portion, 50.75% (34 individuals), falls into the overweight category with a BMI between 23 and 27.5. Additionally, 29.85% (20 individuals) are classified as obese, with a BMI greater than 27.5. This distribution highlights that the majority of the study group is either OW or OB, with only a small percentage maintaining a normal BMI. Table 6 shows that, for Total Cholesterol (TC) levels greater than 200 mg/dL, 32.84% (22 cases) are normal, while 67.16% (45 cases) are elevated. There are no cases with reduced TC levels. For Triglyceride (TG) levels greater than 150 mg/dL, 44.78% (30 cases) are normal, and 55.22% (37 cases) are elevated. There are no cases with reduced TG levels. For High-Density Lipoprotein (HDL) levels below 140 mg/dL, 25.37% (17 cases) are normal, with no elevated cases. A significant 74.63% (50 cases) have reduced HDL levels. For Low-Density Lipoprotein (LDL) levels greater than 130 mg/dL, 34.33% (23 cases) are normal, while 65.67% (44 cases) are elevated. There are no cases with reduced LDL levels.

Table 7 shows that, for individuals aged 18-30, 59.26% (16 cases) have normal TC levels, while 40.74% (11 cases) have elevated TC levels. In the 31-40 age groups, only 25.00% (10 cases) have normal TC levels, whereas a significant 75.00% (30 cases) have elevated (E) TC levels. This data highlight that a higher percentage of E-TC levels are found among the older age group (31-40) compared to the younger age group (18-30). Table 8 shows that, in the 18-30 age groups, 21 cases (77.78%) had normal TG levels, while 7 cases (25.93%) had elevated TG levels. In contrast, in the 31-40 age groups, 11 cases (27.50%) had normal TG levels, and 31 cases (77.50%) had elevated TG levels. These data highlights that a higher percentage of elevated TG levels is found among the older age group (31-40) compared to the younger age group (18-30). Table 9 shows that, for individuals aged 18-30, 26.6% of cases showed normal HDL levels, while 73.3% had reduced HDL levels. In the 31-40 age group, 26.9% exhibited normal HDL levels, contrasting with 73% showing elevated HDL levels. These percentages reflect a higher proportion of reduced HDL levels in both age groups, particularly notable among those aged 31-40. Table 10 shows that, among individuals aged 18-30, 45.45% had normal LDL levels, while 31.11% had elevated LDL levels. In contrast, for those aged 31-40, 54.55% exhibited normal LDL levels, with 68.89% showing elevated LDL levels. These percentages suggest a higher prevalence of elevated LDL levels in older adults compared to younger individuals in this study. Table 11 shows that, among individuals aged 18-30, 29.63% showed ST elevation (ST-E), while 47.50% did not exhibit this change. In contrast, among those aged 31-40, 70.37% presented with ST-E, whereas 52.50% did not. These percentages highlight differing prevalence of ST wave changes across age groups, a higher incidence of ST-E among individuals aged 31-40 compared to those aged 18-30.

Table 12 shows that, among males, 85.19% showed ST-E, while 67.50% did not exhibit this change. For females, 14.81% presented with ST-E, and 32.50% did not. In total, 27 individuals showed ST-E (40.30% of the total), and 40 did not (59.70% of the total) across both genders. These percentages indicate a higher prevalence of ST-E among males compared to females. Table 13 shows that, among those with ST-E (present), the percentages of cases experiencing each symptom like chest pain (70.37%), palpitation (37.04%), sweating (40.74%), giddiness (25.93%), vomiting (59.26%) and AP (29.63%). For individuals without ST-E (absent), the corresponding percentages include chest pain (45.00%), palpitation (32.50%) and sweating (52.50%), giddiness (32.50%), vomiting (50.00%) and abdominal pain (30.00%). Data shows that chest pain is prevalent among those with ST-E, and sweating and vomiting among those without ST-E. Table 14 shows that, an among individuals with a normal BMI, 30.77% had TC levels exceeding 200 mg/dL, 30.77% had TG levels above 150 mg/dL, 76.92% had HDL levels below 40 mg/dL, and 23.08% had LDL levels considered high. For those classified as OW, 55.88% had TC levels greater than 200 mg/dL, 38.24% had elevated TG levels, 52.94% had low HDL levels, and 50.00% had elevated LDL levels. Among individuals categorized as OB, 85.00% had TC levels exceeding 200 mg/dL, 80.00% had elevated (E) TG levels, 85.00% had reduced HDL levels, and 90.00% had E-LDL levels.

## Discussion:

In our study of 67 patients, the majority (59.70%) were aged 31-40 years, while 40.30% belonged to the 18-30 years age group. The mean age of our study population was 32.98 ± 5.45 years. Nagamalesh *et al.* conducted a study on 63 patients, with 33.3% in the 26-35 age group and 66.7% in the 36-45 age group [8]. Males comprised 74.63% and females 25.37% of our study, with a male-to-female ratio of 3:1. Ralapanawa *et al.* (2019) studied 100 ACS patients with a mean age of 35.92 ± 4.45 years, reporting a male-to-female ratio of approximately 2:1 [9]. Among our study participants, 37.31% lead a SL, with 44.00% in the 18-30 age group and 33.33% in the 31-40 age group. The younger age group predominantly leads a SL. Males (42%) are more sedentary compared to females (24%). Family history of CAD was found in 26.87% of individuals, with 41% in the younger age group and 23% in the older age group. Most participants (73.13%) had no family history of CAD. Males (34%) showed a higher prevalence of family history compared to females (18%). Tobacco use was prevalent in 74.63% of participants, with 56% in the 18-30 age group and 88% in the 31-40 age group. Males (80%) had a higher prevalence of tobacco use than females (59%). Alcohol consumption was reported by 62.69% of participants, with 70% in the younger age group and 58% in the older age group. Males (72%) were more likely to consume alcohol than females (35%). In 2020, Cheema and colleagues examined individuals aged 18-40, finding that acute coronary syndrome predominantly impacts males. Key modifiable risk factors identified were hypertension, diabetes mellitus, smoking, and dyslipidemia. They also noted a prevalent non-modifiable risk factor in these patients: a positive family history of ACS [10].

Symptomatically, 58.21% experienced chest pain of which chest pain was prevalent in 52.00% of the younger group and 57.14% of the older group. Similarly, 66.67% of males and 23.08% of females reported this symptom. Palpitation is noted in 34.33%, of which 52.00% belonged to the younger group and 23.81% to the older group. 37.04% of males and 23.08% of females showed this symptom. Jayachandra, Srinivasa and colleagues examined young patients with CAD who showed distinct R/F profiles compared to older patients. Hypertension (HYT) and smoking(SM) were the predominant R/F among the younger cohort, whereas DM, kidney disease (KD) and SM were more prevalent among the elderly patients [11]. In our study, TC levels were elevated in 67.16%, TG levels were elevated in 55.22%, HDL were reduced in 74.63% and LDL levels were elevated in 65.67%. Nagamalesh Um *et al.* found that dyslipidemia (69.8%), DM (42.9%), and HYT (20.6%) were the most prevalent risk factors among their study participants [12]. In 2019, Iragavarapu and colleagues reported that fasting lipid profile tests showed evidence of dyslipidemia in 17.5% of young patients and 9% of elderly patients. This difference was statistically significant in the younger group [13]. Among those with ST-E (present) in this study, the percentages of cases experiencing each symptom are as follows: chest pain (70.37%), palpitation (37.04%), sweating (40.74%), giddiness (25.93%), vomiting (59.26%), AP (29.63%), and chest discomfort (CD) (48.15%). Ralapanawa *et al.* (2018) found that the highest proportion of patients with NSTEMI (27.3%) and unstable angina (32.7%) were in the age groups of 60-69 years. They also noted that the highest percentage of patients with STEMI (35.1%) fell within the age range of 50-59 years [9]. When compared to older patients, very young individuals presented with less significant coronary artery disease (CAD). This is likely attributable to the fact that they had less atherosclerosis in the coronary arteries. Both smoking and dyslipidemia are considered to be the most significant modifiable risk factors in the very young Indian population. Through primary prevention, which involves educating the general population about the consequences of smoking, unhealthy eating habits, and a sedentary lifestyle in the early years of life, it may be possible to avoid the development of heart issues later in life. As a consequence, this will result in a reduction of the pressure that is being placed on our nation's health care system, which is already thinly stretched [14]. Young patients with acute coronary syndrome demonstrate a significant male predominance. We have identified obesity and hypertension as the predominant risk factors, affecting approximately 40% of participants. In contrast, traditional risk factors, including diabetes and hypertension, are notably less common among individuals under 30 years of age when compared to their older counterparts. This young cohort primarily exhibits single-vessel disease, with a statistically significant prevalence of left anterior descending (LAD) artery involvement and a notably reduced incidence of multi-vessel disease when compared to older young patients. Individuals aged over 30 years exhibit a higher prevalence of multiple vessel disease in comparison to their counterparts less than 30 years of age. The results make it clear how important it is to find and deal with risk factors in young adults as soon as possible in order to stop acute coronary syndrome from getting worse and lower the risk of long-term health problems and death [15]. Clinicians in India should take a broader approach to preventing cardiovascular disease by including risk factors that can be changed that are specific to our population. These include a higher waist-to-hip ratio, low HDL cholesterol levels and poorly managed diabetes mellitus. Consequently, it is alarming that there is a significant prevalence of these risk factors within the general population [16]. There is a notable predominance of males, with a mean age of 56 years. Tobacco has been identified as a significant risk factor, accounting for 65%, while obesity, defined as a BMI greater than 25, represents the least common risk factor at 13%. Patients presented with typical chest pain in 94% of cases, and electrocardiogram findings indicated anterior wall changes in 54% of the subjects. Forty percent of patients experienced complications, with the majority being arrhythmias (60%) and the least common being mechanical complications (2.5%). In conclusion, our findings indicate that acute coronary syndrome (ACS) is more prevalent among adult males, with tobacco use identified as a significant risk factor within our population [17].

## Conclusion:

Data shows that modifiable R/F was seen for tobacco use, alcohol consumption and sedentary life. In addition to above, clinical biomarker was found as dyslipidemia.

## Figures and Tables

**Table 1:** Age distribution

**Age groups**	**No of cases**	**Percentage**
(18-30) years	27	40.30%
(31-40) years	40	59.70%

**Table 2:** Gender distribution

**Gender**	**No of cases**	**Percentage**
Male	50	74.63%
Female	17	25.37%

**Table 3:** Clinical feature (C/F)

**Age group**	**18-30 (n=25)**		**18-30 (n=42)**			
**Clinical features**	**No of cases**	**Percentage**	**No of cases**	**Percentage**	**Total**	**P-value**
Sedentary life(SL)	11	44.00%	14	33.33%	25	0.75
Chest pain(CP)	13	52.00%	24	57.14%	39	0.21
Palpitation(PP)	13	52.00%	10	23.81%	23	0.001
Sweating(SW)	14	56.00%	18	42.86%	32	0.66
Giddiness(GD)	6	24.00%	14	33.33%	20	0.4
Vomiting(VM)	10	40.00%	29	69.05%	39	0.03
Abdominal pain(AP)	7	28.00%	13	30.95%	20	0.53

**Table 4:** C/F According to gender

**Gender**	**Male**		**Female**		**Total**	**P-value**
**Clinical features**	**No of cases**	**Percentage**	**No of cases**	**Percentage**		
SL	23	42.59%	2	15.38%	25	0.02
CP	36	66.67%	3	23.08%	39	0.0003
PP	20	37.04%	3	23.08%	23	0.07
SW	27	50.00%	5	38.46%	32	0.017
GD	14	25.93%	6	46.15%	20	0.4
VM	29	53.70%	10	76.92%	39	0.03
AP	15	27.78%	5	38.46%	20	0.29

**Table 5:** BMI Distribution

**BMI**	**No of cases**	**Percentage**
18.5-23 (normal)	13	19.40%
23-27.5 (overweight)(OW)	34	50.75%
> 27.5 (obese)(OB)	20	29.85%

**Table 6:** Lipid profile

**Lipid profile**	**Normal**		**Elevated**		**Reduced**	
	**No of cases**	**Percentage**	**No of cases**	**Percentage**	**No of cases**	**Percentage**
TC> 200	22	32.84%	45	67.16%	0	0.00%
TG> 150	30	44.78%	37	55.22%	0	0.00%
HDL< 140	17	25.37%	0	0.00%	50	74.63%
LDL> 130	23	34.33%	44	65.67%	0	0.00%

**Table 7:** TC level

**TC**	**Normal**		**Elevated**	
**Age group**	**No of cases**	**Percentage**	**No of cases**	**Percentage**
18-30	16	59.26%	11	40.74%
31-40	10	25.00%	30	75.00%

**Table 8:** TG level

**TG**	**Normal**		**Elevated**		**p-value**
**Age group**	**No of cases**	**Percentage**	**No of cases**	**Percentage**	
18-30	21	77.78%	7	25.93%	0.0001
31-40	11	27.50%	31	77.50%	

**Table 9:** HDL

**HDL**	**Normal**		**Elevated**		**p-value**
**Age group**	**No of cases**	**Percentage**	**No of cases**	**Percentage**	
18-30	4	26.60%	14	26.90%	0.001
31-40	11	73.30%	38	73.00%	

**Table 10:** LDL

**LDL**	**Normal**		**Elevated**		**p-value**
**Age group**	**No of cases**	**Percentage**	**No of cases**	**Percentage**	
18-30	10	45.45%	14	31.11%	0.004
31-40	12	54.55%	31	68.89%	

**Table 11:** ST wave change

**ST elevation**	**Present**		**Absent**		**p-value**
**Age group**	**No of cases**	**Percentage**	**No of cases**	**Percentage**	
18-30	8	29.63%	19	47.50%	0.1435
31-40	19	70.37%	21	52.50%	

**Table 12:** ST Wave

**ST elevation**	**Present**		**Absent**		**Total**	**p-value**
**Gender**	**No of cases**	**Percentage**	**No of cases**	**Percentage**		
Male	23	85.19%	27	67.50%	50	
Female	4	14.81%	13	32.50%	17	0.1028
Total	27	100.00%	40	100.00%	67	

**Table 13:** ST wave change

**ST elevation**	**Present (n=27)**		**Absent (n=40)**		**Total**
**Symptoms**	**No of cases**	**Percentage**	**No of cases**	**Percentage**	
CP	19	70.37%	18	45.00%	39
PP	10	37.04%	13	32.50%	23
SW	11	40.74%	21	52.50%	32
GD	7	25.93%	13	32.50%	20
VM	16	59.26%	20	50.00%	39
AP	8	29.63%	12	30.00%	20

**Table 14:** MI Distribution

**Symptoms**	**18.5-23 normal**		**23-27.5 overweight**		**> 27.5 obese**	
	**No.**	**%**	**No.**	**%**	**No.**	**%**
TC>200	4	30.77%	19	55.88%	17	85.00%
TG> 150	4	30.77%	13	38.24%	16	80.00%
HDL< 40	10	76.92%	18	52.94%	17	85.00%
LDL>	3	23.08%	17	50.00%	18	90.00%

## References

[1] Faisal AW (2011). Journal of Ayub Medical College Abbottabad..

[2] Yadav P (2010). National journal of community medicine..

[3] Chan MY (2016). International Journal of Cardiology..

[4] Ralapanawa U, Sivakanesan R (2021). Journal of epidemiology and global health..

[5] Okrainec K (2004). American heart journal..

[6] Beyranvand MR (2011). The quality of life after first acute myocardialinfarction..

[7] Mosa Farkhani E (2014). Medical journal of mashhad university of medical sciences..

[8] Nagamalesh UM (2018). Journal of Clinical and Preventive Cardiology..

[9] Ralapanawa U (2019). BMC cardiovascular disorders..

[10] Cheema FM (2020). Pakistan Journal of Medical Sciences..

[11] Jayachandra S (2014). Heart India..

[12] Nagamalesh UM (2018). Journal of Clinical and Preventive Cardiology..

[13] Iragavarapu T (2019). Journal of the practice of cardiovascular sciences..

[14] Joshi P (2022). Heart India..

[15] Mahorkar A (2024). European Journal of Cardiovascular Medicine..

[16] Bansal M (2023). Indian Journal of Clinical Cardiology..

[17] Yadav P (2010). National journal of community medicine..

